# Behavioral health interventions for pediatric acute lymphoblastic leukemia: a scoping review

**DOI:** 10.3389/fonc.2025.1697894

**Published:** 2025-12-09

**Authors:** Carolyn R. Bates, Sydney M. Gibson, Peter R. Johnson, Sarah L. Hunt, Patrick M. Dyjak, Courtney A. Gibson, Christina M. Sharkey, Kimberly L. Klages

**Affiliations:** 1 Department of Pediatrics, University of Kansas Medical Center, University of Kansas Cancer Center, Kansas City, KS, United States; 2 Kansas City University College of Osteopathic Medicine, Kansas City, MO, United States; 3 A.R. Dykes Library, University of Kansas Medical Center, Kansas City, KS, United States; 4 Department of Psychology, The Catholic University of America, Washington, DC, United States; 5 Division of Behavioral Medicine and Clinical Psychology, Cincinnati Children's Hospital Medical Center, Cincinatti, OH, United States

**Keywords:** acute lymphoblastic leukemia (ALL), pediatric, health behavior, behavioral intervention, behavioral health

## Abstract

**Background:**

Acute Lymphoblastic Leukemia (ALL) is the most common pediatric cancer, with significant advancements in treatment leading to over 90% five-year event-free survival rates in developed countries. However, disruptions in health behaviors during treatment, such as diet, activity, and sleep, can negatively impact treatment tolerance and increase risks of late effects. This scoping review aimed to assess the breadth of existing behavioral health interventions designed to improve immediate and long-term health outcomes for children and adolescents receiving treatment for ALL.

**Method:**

The review focused on behavioral interventions for children (ages 2–18 years) undergoing active treatment for ALL. We followed the JBI Manual of Evidence Synthesis and PRISMA-ScR guidelines, including a comprehensive search across PubMed, Elsevier, and Web of Science databases. Articles were screened, assessed, and data extracted by multiple reviewers to identify behavioral health promotion interventions used during ALL treatment.

**Results:**

A total of 157 full-text articles were screened, and 86 met inclusion criteria. Most interventions targeted physical activity and exercise (n=36), procedural distress (n=24), nutrition (n=8), and symptom reduction/management (n=6), with others targeting pain, sleep, and health-related quality of life. Date of publication, methodology, and behavioral intervention components varied considerably across studies.

**Conclusions:**

The literature on behavioral health promotion interventions during pediatric ALL treatment is broad but lacks depth, with many studies limited by small sample sizes and stalling at the feasibility stage. There is a need for larger, more rigorous trials to assess the effectiveness of these interventions and improve outcomes for youth with ALL.

## Introduction

Acute Lymphoblastic Leukemia (ALL) is the most prevalent childhood cancer with an estimated 6,660 new cases annually (1). Treatment advancements have dramatically improved outcomes for children with ALL, with five-year event-free survival rates now reaching 90% for children and 74% for adolescents in developed countries (2). As survival rates have increased, there has been a growing recognition of the importance of behavioral health factors during treatment that may influence both immediate outcomes and long-term survivorship (3). Disruptions in health behaviors, including diet, activity, and sleep, are common during ALL treatment and can lead to lower treatment tolerance, increased toxicity and heightened risk for late effects, morbidity, and mortality (4).

To improve health outcomes in children undergoing ALL treatment, interventions have been developed to target a range of behavioral health concerns. Behavioral interventions are nonpharmacological approaches aimed at improving health outcomes by targeting modifiable behaviors (e.g., physical activity, sleep, nutrition, coping skills) and psychosocial functioning through structured activities, education, or therapeutic techniques. Behavioral intervention targets vary in focus and originate from diverse disciplines, including dietetics, physical therapy, psychology, and nursing. However, the existing literature is highly fragmented, with studies dispersed across discipline-specific journals, making it difficult to synthesize findings and translate them into clinical practice (5–7). Furthermore, most existing behavioral interventions target a single health outcome, such as physical functioning (8–14) or fatigue (11, 15, 16), or health behavior, such as sleep (17) or diet (18, 19) which further complicate efforts to synthesize the literature, given the wide range of distinct behavioral targets and the lack of integrated, multi-component approaches.

Given the breadth and heterogeneity of this literature, a scoping review is warranted to systematically identify, categorize, and map behavioral health promotion interventions implemented during pediatric ALL treatment. Scoping reviews are particularly appropriate for topics with diverse and emerging evidence bases, where a broad overview is needed to clarify key concepts, highlight knowledge gaps, and inform future research and clinical practice. The purpose of this review was to examine the range of behavioral health promotion interventions designed to improve health outcomes for children and adolescents receiving treatment for ALL. The primary review questions were:

1. What behavioral interventions have been implemented with children on active ALL treatment?
2. What health behavior outcomes are targeted?
3. Are these interventions used independently or in combination with other intervention(s)?

i. What component(s) are utilized in each intervention?

## Methods

A scoping review was developed around the concepts of 'acute lymphoblastic leukemia,' ‘children’ (ages 2-18), and behavioral interventions during active cancer treatment including: behavior therapy, exercise therapy, movement-based therapy, physical therapy, nutrition/diet therapy, cognitive behavioral therapy, relaxation therapy, and muscle stretching therapy. This review was conducted following guidance in the Joanna Briggs Institute (JBI) Manual of Evidence Synthesis and reported according to the Preferred Reporting Items for Systematic reviews and Meta-Analyses (PRISMA) extension for scoping reviews (PRISMA-ScR). Following the creation and registration of a reporting protocol (20) in Open Science Framework (OSF) an initial search aligned with the Population, Concept, and Context of the review was drafted for PubMed using appropriate Medical Subject Headings (MeSH) terms and keywords by a health sciences librarian (PJ) in consultation with the review team. This search was then reviewed by another health sciences librarian following the Peer Review of Electronic Search Strategies (PRESS) criteria (21). Recommended changes were applied, and the search was translated to the databases Embase.com (Elsevier) and Web of Science Core Collections - SCI-Expanded 1900-present, SSCI 1956-present, AHCI 1975-present, ESCI 2020-present (Clarivate) using comparable keywords and Emtree terms. No database filters or limits were applied to the search. The references of any literature or systematic reviews identified in the search were screened independently in a hand searching process and added to the title/abstract screening stage. The full search strategy and its translations along with a PRISMA-ScR checklist are available as supplemental material for this review. 

Citation files from each database and hand searching results were exported to EndNote 21 (Clarivate) for management/retraction watching and then into Covidence (Melbourne, VIC, Australia) for deduplication, screening, assessment, and data extraction. In Covidence, two reviewers independently screened each included article according to the inclusion/exclusion criteria, and a third resolved any conflict. Published quantitative, qualitative, and case studies were included if (1) they were original research, (2) they included a health behavior intervention, (3) the sample included patients with ALL (2–18 years of age) who were actively receiving treatment with curative intentions, and (4) the article was available in English. Relevant studies investigating a mixture of diagnoses, such as both ALL and other pediatric oncology diagnoses, were included only if ALL diagnoses were represented in the sample and all other inclusion criteria were met. Articles that evaluated health behavior interventions in survivors of pediatric ALL or patients receiving hospice care were excluded. Books and book chapters, dissertations, editorial letters, conference abstracts and articles not available in English were also excluded. While systematic reviews and meta-analyses were not included in this review, these articles and reference lists were evaluated to identify relevant original publications. Screened articles matching the inclusion criteria were added to full text assessment, and full text copies were obtained through library subscriptions or interlibrary loan. Assessed articles matching the inclusion criteria were added to the extraction stage. A data extraction form was developed within Covidence matching inclusion criteria. The form was piloted on three random studies from the extraction pool and modified before being applied to the entire pool. Two reviewers independently extracted data from each article and when disagreements between coders occurred, a third reviewer provided consensus. Data extracted from each article included study design, country of origin, age range, patient diagnoses included (i.e., ALL only, mixed sample of ALL and other cancers, undefined leukemia), cancer treatment type (i.e., active chemotherapy, radiation therapy, stem cell therapy, treatment not specified), total number of participants, intervention target (i.e., physical activity, procedural distress, pain, health-related quality of life/psychological functioning, nutrition/dietary, sleep, and symptom reduction), intervention type (i.e., education, physical activity/exercise promotion, coaching/supportive care , coping strategies, cognitive behavioral therapy, complementary/alternative medicine [CAM]), person receiving intervention (i.e., patient, caregiver), intervention setting, mode of delivery, and outcomes measured. Extracted data was exported out of Covidence as a.csv file for charting in Excel and analysis.

## Results

**Figure 1:**
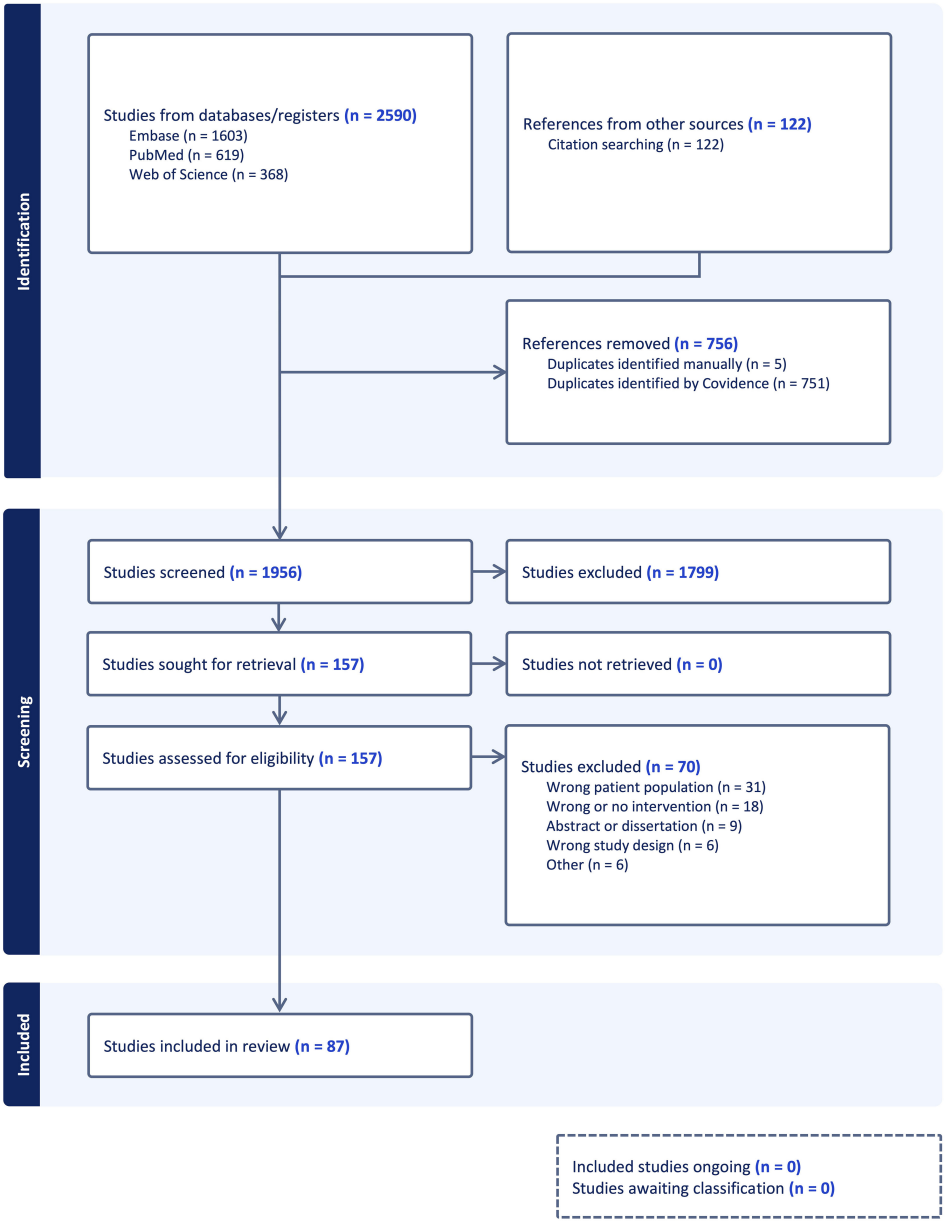
PRISMA flow diagram.

This scoping review included a total of 86 studies describing behavioral health promotion interventions delivered during pediatric ALL treatment (**Figure 1**). Interventions varied by behavioral target, modality, delivery format, and developmental age group. Findings are summarized below by behavioral domain, with emphasis on intervention types, populations, delivery settings, and areas for future research. See **Table 1** for a summary of included intervention studies.

**Table 1:** Included Studies.

**Reference**	**Country**	**Behavioral Domain**	**Study Design**	**Diagnoses Included**	**Cancer Treatment**	***N***	**Age Range (years)**	**Intervention Type**	**Outcome(s) Measured**
Alemi et al., 2016 (22)	Iran	HRQoL/ Psychological functioning	Non-RCT	Mixed sample	Active chemotherapy	11	7-12	CBT; Other: robot assisted therapy	Psychological distress
Barbieri et al., 2021 (23)	Italy	Physical activity/ Exercise	Cohort; Feasibility	Mixed sample	Active chemotherapy; Stem cell therapy	104	3-19	Physical activity	Functional disability; Range of motion; Posture, Mobility, Cranial-sacral rhythmic impulse
Barrera, 2000 (24)	Canada	Procedural distress	Case report	ALL only	Active chemotherapy	1	4	CBT	Psychological distress; Procedural distress
Beulertz et al., 2016 (25)	Germany	Physical activity/ Exercise	Non-RCT	Mixed sample	Active chemotherapy; Radiation; Other: surgery	53	4-17	Physical activity	Physical activity; HRQoL; Functional disability
Bogg et al., 2015 (26)	Australia	Physical activity/ Exercise	Feasibility; Pilot	Mixed sample	Stem cell therapy	14	6-18	Physical activity	Physical activity; HRQoL; Fatigue
Broome et al., 1992 (27)	USA	Procedural distress	Case series	ALL only	Active chemotherapy	14	3-15	Coaching/Supportive care; Coping strategies: relaxation	Pain; Psychological distress; Procedural distress
Broome et al., 1998 (28)	USA	Procedural distress	Single-arm	Mixed sample	Not specified	19	4-18	Coping strategies: relaxation	Pain; Procedural distress
Chamorro-Viña et al., 2010 (29)	Spain	Physical activity/ Exercise	Non-RCT	Mixed sample	Stem cell therapy	20	<16	Physical activity	Physical activity; Anthropometry/Body composition; BMI; Symptom presence
Chen et al., 1999 (30)	USA	Procedural distress	RCT	ALL only	Active chemotherapy	50	3-18	Coping strategies: cognitive modification	Pain; Psychological distress; Procedural distress
Chen et al., 2000 (31)	USA	Procedural distress	RCT	ALL only	Active chemotherapy	55	3-18	Coping strategies: cognitive modification	Pain; Psychological distress; Procedural distress
Cox et al., 2018 (8)	Canada; USA	Physical activity/ Exercise	RCT	ALL only	Active chemotherapy	73	4-19	Physical activity; Coaching/Supportive care	Physical activity; BMI; HRQoL; Pain; Functional disability
Dalla Santa et al., 2023 (32)	Australia	Physical activity/ Exercise	Single-arm; Feasibility	Mixed sample	Active chemotherapy	14	4-18	Physical activity	Physical activity; HRQoL; Symptom presence
Esbenshade, 2014 (33)	USA	Physical activity/ Exercise	Single-arm; Feasibility; Pilot	ALL only	Active chemotherapy	17	5-10	Physical activity; Coaching/Supportive care	BMI; Functional disability; Motor skills; Flexibility; Strength
Gaser et al., 2022 (34)	Germany	Physical activity/ Exercise	RCT	Mixed sample	Active chemotherapy; Radiation; Stem cell therapy	41	4-18	Physical activity	Physical activity; Functional disability
Gershon et al., 2004 (35)	USA	Procedural distress	Feasibility; Pilot	Mixed sample	Active chemotherapy	59	7-19	Coping strategies: distraction; Other: virtual reality	Pain; Procedural distress
Geyer et al., 2011 (36)	USA	Physical activity/ Exercise	Feasibility	Mixed sample	Active chemotherapy; Stem cell therapy	6	5-19	Coping strategies: relaxation; CAM: yoga	HRQoL
Ghaffar et al., 2019 (37)	Pakistan	Nutrition/ Dietary	Non-RCT	ALL only	Not specified	30	5-15	Eating/Feeding	Dietary; Anthropometry/Body composition; Blood serum values
Gohar et al., 2011 (9)	USA	Physical activity/ Exercise	Feasibility	ALL only	Active chemotherapy	9	2-18	Physical activity	HRQoL; Functional disability
Gupta et al., 2022 (38)	India	Nutrition/ Dietary	RCT; Pilot	Mixed sample	Active chemotherapy	42	3-14	Eating/Feeding	Dietary; Symptom presence
Guru et al., 2023 (39)	India	Nutrition/ Dietary	Single-arm	Mixed sample	Not specified	455	<18	Eating/Feeding	Dietary; Anthropometry/Body composition
Hartman et al., 2009 (10)	The Netherlands	Physical activity/ Exercise	RCT	ALL only	Active chemotherapy	51	1-18	Physical activity	Physical activity; Functional disability
Hill et al., 2018 (18)	USA	Nutrition/ Dietary	Cohort	ALL only	Active chemotherapy	67	1-20	Eating/Feeding	BMI
Hooke et al., 2016 (11)	USA	Physical activity/ Exercise	Single-arm; Feasibility; Pilot	ALL only	Active chemotherapy	16	6-18	Physical activity; Coaching/Supportive Care	Physical activity; Fatigue
Hooke et al., 2019 (40)	USA	Physical activity/ Exercise	Non-RCT; Pilot	Mixed sample	Active chemotherapy	30	6-18	Physical activity; Coaching/Supportive care	Physical activity; Fatigue
Hsiao et al., 2019 (41)	Taiwan	Procedural distress	Pilot	Mixed sample	Active chemotherapy	18	3-11	Coping skills: distraction, relaxation	Procedural distress
Jacknow et al., 1994 (42)	USA	Symptom reduction/ management	RCT	Mixed sample	Active chemotherapy	20	6-18	CAM: hypnosis	Symptom presence; Pain medication usage
Jay et al., 1987 (43)	USA	Procedural distress	RCT	Undefined leukemia	Not specified	56	3-13	CBT; Other: diazepam	Pain; Procedural distress
Jay et al., 1991 (44)	USA	Procedural distress	RCT	Mixed sample	Not specified	83	3.5-12	CBT	Pain; Procedural distress
Jibb 2017 (45)	Canada	Pain	Feasibility; Pilot	Mixed sample	Active chemotherapy; Radiation; Stem cell therapy; Other: surgery	40	12-18	Coaching/Supportive care	HRQoL; Pain
Jibb et al., 2018 (46)	Canada	Procedural distress	RCT; Feasibility; Pilot	Mixed sample	Active chemotherapy; Radiation; Other: surgery	40	4-9	Coping skills: distraction, relaxation	Pain; Procedural distress
Kazak et al., 1996 (47)	USA	Procedural distress	RCT	Mixed sample	Active chemotherapy	162	<18	Eating/Feeding; Coping skills: distraction, relaxation	HRQoL; Psychological distress; Procedural distress
Kazak et al., 1998 (48)	USA	Procedural distress	RCT; Feasibility; Pilot	ALL only	Active chemotherapy	122	<18	Coping skills: distraction, relaxation	HRQoL; Procedural distress
Keats et al., 2008 (49)	Canada	Physical activity/ Exercise	Feasibility; Pilot	Mixed sample	Active chemotherapy; Radiation; Stem cell therapy	10	14-19	Physical activity	Physical activity; BMI; HRQoL; Fatigue
Kellerman, 1979 (50)	USA	Sleep	Case report	ALL only	Active chemotherapy	1	3	Sleep	Sleep; Number of night terrors
Kellerman et al., 1983 (51)	USA	Procedural distress	Pilot	Mixed sample	Not specified	16	Not reported	CAM: hypnosis	Pain; Psychological distress; Procedural distress
Kemper et al., 2009 (52)	USA	Pain	Pilot	Mixed sample	Not specified	9	Not reported	CAM: healing touch	Pain; Heart rate variability
Kesting et al., 2022 (53)	Germany	Physical activity/ Exercise	Feasibility; Pilot	Mixed sample	Active chemotherapy; Radiation; Other: surgery	11	6-18	Physical activity	Physical activity
Kirizawa et al., 2021 (54)	Brazil	Physical activity/ Exercise	Case-control	Undefined leukemia	Active chemotherapy	21	4-14	Exercise; CAM: biofeedback	Blood pressure; Respiratory rate; Heart rate variability
Knoerl et al., 2022 (55)	USA	HRQoL/ Psychological functioning	Single-arm; Feasibility; Pilot	Mixed sample	Active chemotherapy	27	15-39	Other: mindfulness, music therapy	Psychological distress
Kolko et al., 1985 (56)	USA	Symptom reduction/ management	Single-arm; multiple baseline; ABAB	ALL only	Active chemotherapy	3	11-17	Coping skills: distraction	Pain; Psychological distress; Procedural distress; Symptom presence
Kuttner et al., 1988 (57)	Canada	Procedural distress	RCT	Undefined leukemia	Not specified	48	3-10	Coping skills: distraction; CAM: hypnosis	Pain; Psychological distress; Procedural distress
Lam et al., 2018 (58)	China	Physical activity/ Exercise	RCT	Mixed sample	Active chemotherapy; Other: surgery	70	9-18	Physical activity; Coaching/Supportive care	Physical activity; HRQoL; Fatigue
Lebaron et al., 1984 (59)	USA	Symptom reduction/ management	Pilot	Mixed sample	Active chemotherapy	8	10-17	Coping skills: distraction	Symptom presence
Li et al., 2011 (60)	China	HRQoL/ Psychological functioning	Non-RCT	Mixed sample	Active chemotherapy	122	8-16	Other: virtual reality, medical play	Psychological distress
Li et al., 2017 (61)	USA	Nutrition/ Dietary	RCT	ALL only	Active chemotherapy	22	7-18	Eating/Feeding	Dietary; Anthropometry/Body composition; Oxidative stress
Liossi et al., 1999 (62)	UK	Procedural distress	RCT	Undefined leukemia	Not specified	30	5-15	CBT; CAM: hypnosis	Pain; Procedural distress
Liossi et al., 2007 (63)	UK; Greece	Procedural distress	Single-arm	Mixed sample	Active chemotherapy	45	7-16	CBT	Dietary; Pain; Psychological distress; Procedural distress
Lucia et al., 2005 (64)	Spain	Physical activity/ Exercise	Single-arm; Pilot	ALL only	Active chemotherapy	7	<10	Physical activity	Physical activity; HRQoL; Functional disability
Manne et al., 1994 (65)	USA	Procedural distress	Single-arm	Mixed sample	Not specified	35	3-10	Coaching/Supportive care; Coping skills: distraction, relaxation	Procedural distress
Marchese et al., 2004 (12)	USA	Physical activity/ Exercise	RCT	ALL only	Active chemotherapy	28	4-18	Physical activity	Anthropometry/Body composition; HRQoL; Functional disability
McCarthy et al., 1998 (66)	USA	Procedural distress	Pilot	Undefined leukemia	Not specified	10	3-15	Coaching/Supportive care; Coping skills: distraction, relaxation	Pain; Psychological distress; Procedural distress
McGrath et al., 1986 (67)	Canada	Procedural distress	Pilot	Mixed sample	Active chemotherapy	14	3-14	Coaching/Supportive care; Coping skills: relaxation; CAM: hypnosis	Pain; Psychological distress
Meeks et al., 2022 (68)	USA	Pain	Case report	ALL only	Active chemotherapy	1	15	Physical activity; Symptom management; CBT; CAM: hypnosis, mindfulness	Pain
Moody et al., 2010 (69)	USA	Pain	Case series	Mixed sample	Not specified	20	11-26	CAM: yoga	Pain; Psychological distress
Morales et al., 2020 (70)	Spain	Physical activity/ Exercise	Non-RCT; Cohort	Mixed sample	Active chemotherapy	169	4-18	Eating/Feeding; Physical activity	BMI; Survival; Relapse; Days of hospitalization; Cardiac functioning
Moyer-Mileur et al., 2009 (19)	USA	Physical activity/ Exercise	RCT; Pilot	ALL only	Active chemotherapy	13	4-10	Eating/Feeding; Physical activity	Dietary; Physical activity; Anthropometry/Body composition; BMI; Muscle mass
Napartuk et al., 2023 (71)	Canada	Nutrition/ Dietary	Single-arm	Mixed sample	Active chemotherapy; Radiation	36	1-17	Eating/Feeding	Dietary; Anthropometry/Body composition
Nielsen et al., 2018 (72)	Denmark	Physical activity/ Exercise	RCT	Mixed sample	Active chemotherapy; Radiation	75	6-18	Physical activity; Coaching/Supportive care	Physical Activity; Functional disability; Feasibility/Acceptability
Orgel et al., 2021 (73)	USA	Nutrition/ Dietary	Non-RCT	ALL only	Active chemotherapy	120	10-21	Eating/Feeding; Physical activity	Dietary; Physical activity; Anthropometry/Body composition; BMI; Blood serum values
Pederson, 1996 (74)	USA	Procedural distress	Non-RCT	ALL only	Not specified	8	6-18	Coping skills: distraction, relaxation	Pain; Psychological distress; Procedural distress
Perondi et al., 2012 (13)	Brazil	Physical activity/ Exercise	Single-arm; Pilot	ALL only	Active chemotherapy	6	5-18	Physical activity	Physical activity; HRQoL; Fatigue
Platschek et al., 2017 (75)	Germany	Physical activity/ Exercise	Pilot	Mixed sample	Active chemotherapy	9	6-18	Physical activity	Physical activity; Fatigue; Psychological distress
Post-White et al., 2009 (76)	USA	Pain	Feasibility; Pilot	Mixed sample	Active chemotherapy	50	1-18	CAM: healing touch	Pain; Fatigue; Psychological distress; Symptom presence
Powers et al., 1993 (77)	USA	Procedural distress	Single-arm; Pilot	ALL only	Active chemotherapy	4	3-5	Coping skills: distraction, relaxation	Procedural distress
Rohan et al., 2020 (78)	USA	HRQoL/ Psychological functioning	Case report	ALL only	Active chemotherapy	1	5	Medication adherence	Medication adherence
Rosenhagen et al., 2011 (79)	Germany	Physical activity/ Exercise	Feasibility; Pilot; Case-control	Mixed sample	Stem cell therapy	23	Not reported	Physical activity	Physical activity; HRQoL; Fatigue; Strength; Endurance
Sander Wint et al., 2002 (80)	USA	Pain	RCT; Pilot	Mixed sample	Not specified	30	10-19	Coping skills: distraction	Pain
San Juan et al., 2007 (81)	Spain	Physical activity/ Exercise	Single-arm; Feasibility; Pilot	ALL only	Active chemotherapy	7	4-7	Physical activity	Physical activity; Functional disability; Feasibility/Acceptability
San Juan et al., 2007 (82)	Spain	Physical activity/ Exercise	Feasibility; Pilot	ALL only	Active chemotherapy	7	4-7	Physical activity	Physical activity; Anthropometry/Body composition; HRQoL; Functional disability; Feasibility/Acceptability; Strength
San Juan et al., 2008 (83)	Spain	Physical activity/ Exercise	Non-RCT; Feasibility; Pilot	Mixed sample	Stem cell therapy	16	8-16	Physical activity	Physical activity; Anthropometry/Body composition; HRQoL; Functional disability
Savio et al., 2007 (84)	Italy	Physical activity/ Exercise	Feasibility	Mixed sample	Active chemotherapy; Stem cell therapy	46	0-21	Physical activity	Feasibility/Acceptability
Schneider et al., 1999 (85)	USA	Symptom reduction/ management	Single-arm	Mixed sample	Active chemotherapy	11	10-17	Coping skills: distraction	Psychological distress; Symptom presence
Shore et al., 1999 (86)	Canada	Physical activity/ Exercise	Case series	Mixed sample	Active chemotherapy	6	13-14	Physical activity	Anthropometry/Body composition; Psychological distress; Blood serum values
Slifer et al., 1994 (87)	USA	Procedural distress	Pilot	Mixed sample	Radiation therapy	10	3-7	Other: applied behavioral analysis	Procedural distress; Anesthesia usage
Speyer et al., 2010 (88)	France	Physical activity/ Exercise	RCT	Mixed sample	Active chemotherapy	30	5-18	Physical activity	HRQOL
Tanir et al., 2013 (89)	Turkey	Physical activity/ Exercise	RCT	ALL only	Not specified	40	8-12	Physical activity; Coaching/Supportive care	Physical activity; HRQoL; Functional disability; Blood serum values
Tanner et al., 2017 (90)	USA	Physical activity/ Exercise	Feasibility; Pilot	ALL only	Active chemotherapy	135	1-22	Physical activity	Physical activity; Functional disability
Van Dijk-Lokkart et al., 2016 (91)	The Netherlands	HRQoL/ Psychological distress	RCT	Mixed sample	Active chemotherapy; Radiation	68	8-18	Physical activity; CBT	HRQoL; Psychological distress
Vercher et al., 2016 (92)	USA	Physical activity/ Exercise	Case report	ALL only	Active chemotherapy	1	3	Physical activity	Physical activity; HRQoL; Pain; Functional disability
Walters et al., 2021 (93)	USA	Nutrition/ Dietary	Single-arm; Feasibility; Pilot	ALL only	Active chemotherapy	23	5-21	Eating/Feeding; Coaching/Supportive care	Dietary; BMI; Feasibility/Acceptability
Yeh et al., 2011 (16)	Taiwan	Physical activity/ Exercise	Non-RCT; Feasibility; Pilot	ALL only	Active chemotherapy	Not reported	<18	Physical activity	Physical activity; Fatigue
Zardo et al., 2022 (94)	Italy	Physical activity/ Exercise	Cohort; Feasibility; Case-control	Mixed sample	Active chemotherapy; Stem cell therapy	97	7-19	Physical activity	Physical activity; Functional disability; Strength
Zeltzer et al., 1982 (95)	USA	Procedural distress	RCT	Mixed sample	Not specified	33	6-17	Coping skills: distraction; CAM: hypnosis	Pain; Psychological distress; Procedural distress
Zeltzer et al., 1983 (96)	USA	Symptom reduction/ management	Single-arm	Mixed sample	Active chemotherapy	9	12-20	CAM: hypnosis	Psychological distress; Symptom presence
Zeltzer et al., 1984 (97)	USA	Symptom reduction/ management	RCT	Mixed sample	Active chemotherapy	9	6-17	Coping skills: distraction; CAM: hypnosis	Symptom presence
Zupanec et al., 2017 (98)	Canada	Sleep	RCT; Feasibility; Pilot	ALL only	Active chemotherapy	20	4-10	Sleep	Sleep; Fatigue

HRQoL , health-related quality of life; RCT , randomized control trial; Non-RCT, non-randomized experimental trial; cohort , cohort study; single arm , single arm trial; ALL , acute lymphoblastic leukemia; mixed sample , children with ALL and other cancers; CBT , cognitive behavioral therapy; CAM , complementary and alternative medicine; BMI , body mass index.

### Physical activity/exercise

Thirty-six studies reported on interventions targeting physical activity or exercise. Most focused on structured physical activity (n=34 studies), incorporating aerobic, strength, or mobility training. Additional components included coaching/supportive care (n=7), relaxation (n=1), biofeedback (n=1), and eating/feeding strategies (n=2). Common study designs included pilot/feasibility studies (n=10), single-arm trials (n=6), randomized controlled trials (RCTs; n=9), and non-randomized trials (n=6). Nearly all studies were prospective (n=35), with the majority conducted in the United States (n=10), Spain (n=6), and Germany (n=5). Interventions were primarily delivered in-person during active chemotherapy (n=31), often involving mixed samples of children with ALL and other cancers (n=20). Participant ages ranged from 1 to 22 years. Frequently assessed outcomes included physical activity, functional disability, and health-related quality of life (HRQoL), with additional measures such as body mass index (BMI), anthropometry/body composition, strength, motor skills, posture, endurance, and cardiac or respiratory functioning.

### Procedural distress

Twenty-three studies evaluated interventions aimed at reducing procedural distress (e.g., pain or anxiety during lumbar puncture or port access). Cognitive-behavioral therapy (CBT) components, such as distraction, imagery, and relaxation, were most common (n=18). Other modalities included hypnosis (n=3), virtual reality-based distraction (n=2), and behavioral training for motion control (n=1). One study compared a combined pharmacologic and CBT intervention to standard oncology treatment. Coaching/supportive care was included in four studies. Study designs comprised RCTs (n=11), pilot/feasibility studies (n=5), single-arm trials (n=4), non-randomized experimental studies (n=1), case reports (n=1), and case series (n=1). All studies were prospective, with most conducted in the United States (n=17). Interventions were delivered in-person during outpatient visits (n=11) or hospital admissions (n=8), typically involving mixed samples of children with ALL and other cancers (n=13), most of whom were undergoing active chemotherapy (n=12). Participant ages ranged from 3 to 18 years. Common outcomes included pain, procedural distress, psychological distress, and anesthesia usage.

### Pain

Five studies addressed non-procedural pain using behavioral interventions. These included healing touch (n=2; e.g., massage, Reiki), yoga (n=1), CBT (n=1), and a multidisciplinary pain treatment program combining physical (e.g., exercise) and psychological (e.g., CBT, mindfulness) components (n=1). All studies were conducted in North America and used case reports (n=2) or pilot/feasibility designs (n=3). Interventions were delivered individually and in-person within medical settings. All participants were undergoing active chemotherapy, with most samples including children diagnosed with various cancers including ALL (n=4). Participant ages ranged from 11 to 26 years. Assessed outcomes included pain, HRQoL, psychological distress, and heart rate variability.

### Health-related quality of life/psychological functioning

Five studies targeted HRQoL or psychological functioning. Interventions included CBT (n=2), one paired with physical activity and another with robot-assisted therapy; mindfulness/music therapy (n=1); virtual reality/medical play (n=1); and medication adherence support (n=1). Most interventions were delivered in-person (n=4), with one offering remote access. Participants ranged in age from 5 to 39 years and typically had mixed cancer diagnoses (n=4). Study designs included case reports (n=1), non-randomized experimental studies (n=2), single-arm feasibility/pilot trials (n=1), and RCTs (n=1). Studies were conducted in the United States (n=2), Iran (n=1), The Netherlands (n=1), and China (n=1), with all participants undergoing active chemotherapy. Assessed outcomes included HRQoL, psychological distress, and medication adherence.

### Nutrition/dietary

Eight studies targeted dietary behavior, primarily through nutrition education and goal setting. Two interventions integrated additional behavioral approaches, combining nutrition with physical activity (n=1) or supportive care (n=1). Study designs included cohort studies (n=1), non-randomized experimental studies (n=2), single-arm trials (n=3), and RCTs (n=2). Most interventions were delivered individually and in-person (n=8), with two studies offering remote options. Delivery settings included outpatient (n=8), inpatient (n=5), and home-based formats (n=3). Studies were conducted in the United States (n=4), India (n=2), Pakistan (n=1), and Canada (n=1). Participants were primarily caregivers of children with ALL (n=6), ranging from 1 to 21 years of age. Most studies focused on managing nutritional goals during active treatment (n=6). Commonly assessed outcomes included dietary intake, BMI, and anthropometry/body composition.

### Sleep

Two studies targeted sleep behaviors, both caregiver-focused. One employed sleep hygiene techniques, and the other used relaxation strategies. Study designs included a feasibility pilot RCT and a case report. Both studies were conducted in North America, delivered in outpatient settings, with one offering remote access. Samples involved children with ALL undergoing active treatment, ranging in age from 3 to 10 years. Assessed outcomes included sleep quality, number of night terrors, and fatigue.

### Symptom reduction/management

Six studies addressed symptoms beyond pain, including nausea, neuropathy, and mucositis. Interventions included hypnosis (n=3), distraction (n=4), and virtual reality (n=2). Most studies were conducted in the United States and delivered individually and in-person, during inpatient (n=3) and/or outpatient care (n=3). Interventions primarily targeted patients with ALL and other cancer diagnoses (n=5), all undergoing active chemotherapy (n=6). Participant ages ranged from 6 to 20 years. Commonly assessed outcomes included symptom presence and psychological distress.

### Summary of mapped evidence across domains

Across all behavioral intervention targets, interventions were most frequently conducted in high-income countries (especially the United States), delivered in-person, and included samples of children receiving chemotherapy treatment, with relatively fewer interventions tailored specifically to ALL. Physical activity and procedural distress were the most frequently addressed domains and represented the most methodologically advanced areas, with the highest proportion of RCTs and multidisciplinary interventions. In contrast, interventions for pain, sleep, nutrition, psychological functioning, and symptom management were less common and largely limited to early-stage or descriptive studies (e.g., case reports, pilot/feasibility trials). Caregiver involvement was more prominent in dietary and sleep-related interventions. Few interventions spanned multiple domains or used integrated, biopsychosocial models of care. Gaps were especially evident in digital/remote delivery, interventions for adolescents and young adults, and studies conducted outside North America and Europe. This mapping highlights opportunities to expand behavioral health research across underrepresented symptom domains, populations, and delivery modalities.

## Discussion

This scoping review identified a wide range of behavioral interventions that have been implemented during pediatric ALL treatment to date. Interventions most frequently targeted physical activity/exercise and procedural distress, with common strategies including structured physical activity, distraction, relaxation, and other CBT techniques. These two areas also accounted for the highest number of RCTs, indicating they are the most methodologically developed within this population. However, the majority of studies were limited by small sample sizes, and many interventions remained at the feasibility or pilot stage without progressing to full-scale efficacy testing. Consequently, only 31% of studies reviewed included RCTs, reflecting the early and fragmented state of behavioral intervention research in this field.

**Figure 2:**
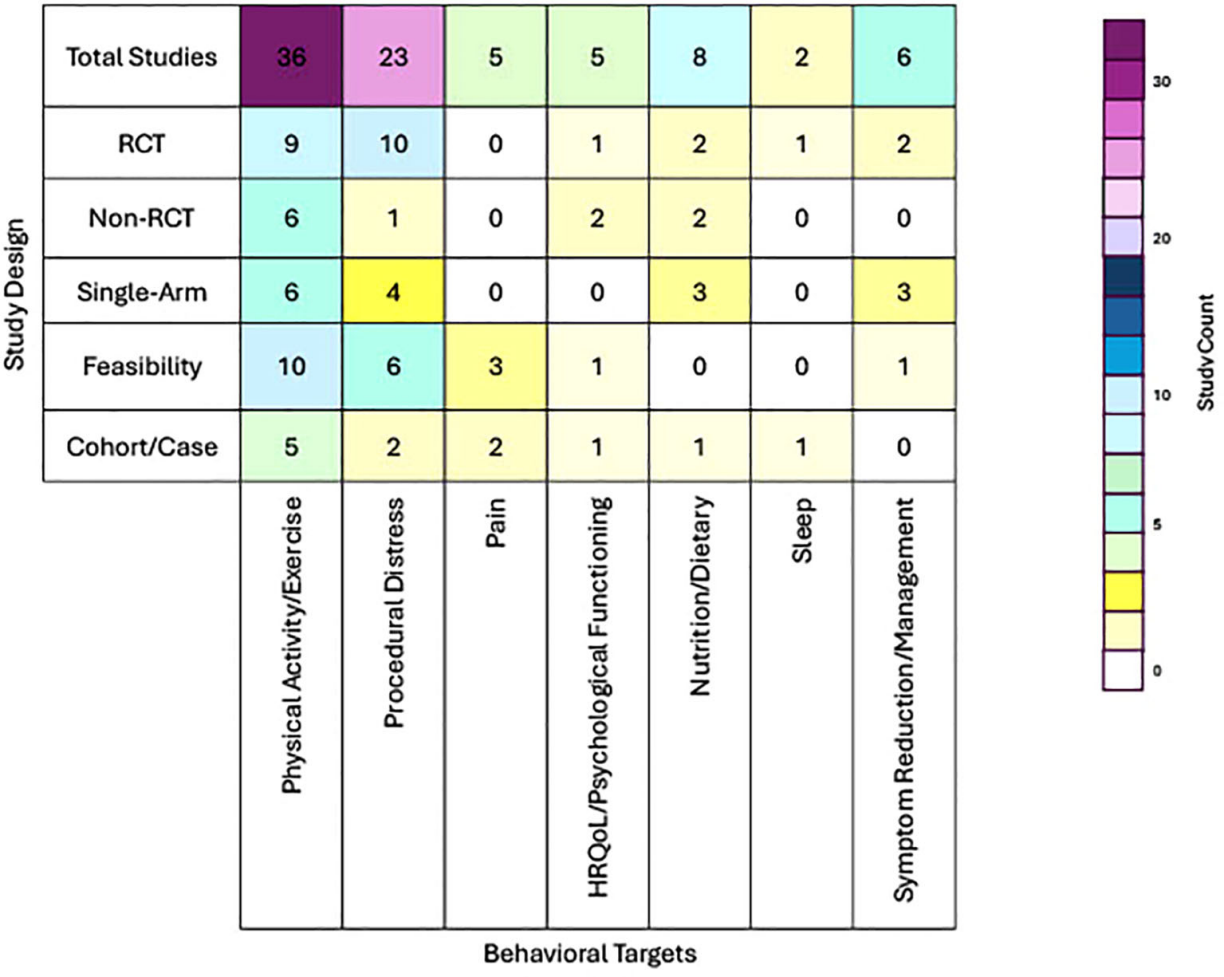
Visual map of study designs across behavioral intervention targets.

Our visual map (**Figure 2**) underscores the uneven distribution of evidence across behavioral domains, intervention types, and delivery settings. For instance, while some domains (e.g., physical activity/exercise) show both high volume and methodological advancement (i.e., more RCTs), others, including sleep, non-procedural pain, and HRQoL/psychological functioning, remain understudied and underdeveloped. In particular, the sleep domain was represented by only two studies, both small in scale and early in design (a pilot feasibility RCT and a case report), despite the well-established role of sleep in recovery and treatment response in pediatric cancer patients. Similarly, the HRQoL/psychological functioning domain, while conceptually broad, lacked a unified focus in the literature and included a small number of heterogeneous studies with mixed samples and intervention formats.

Several of these underrepresented domains were primarily addressed through caregiver-targeted interventions, particularly in the nutrition/dietary and sleep areas. These studies often focused on education or coaching and were typically delivered in outpatient or home-based settings. While promising in terms of feasibility, few of these interventions were rigorously tested or evaluated for their downstream impact on child outcomes, such as nutritional status or sleep quality. Likewise, psychological functioning interventions occasionally incorporated novel elements—such as artificial intelligence or music therapy—but their variability in content and delivery hindered cross-study comparisons and limited conclusions about efficacy or scalability. These findings underscore a need for more systematic development and testing of behavioral interventions across underexplored domains.

Many interventions reviewed in the current study were published over a decade ago, particularly those targeting procedural distress and symptom reduction/management. Notably, 89% of procedural distress interventions were more than 10 years old, and 68% were over 20 years old; all symptom management interventions were published more than 20 years ago. This striking lack of recent research highlights a significant lag in behavioral health intervention development compared to advances in medical treatment and technology. For example, procedural management, such as standardized sedation, has evolved significantly in the last 10 years (97). Yet, despite growing evidence for modern nonpharmacological modalities, such as virtual reality, mindfulness, and acceptance-based interventions, these approaches remain underrepresented in the literature (99). Instead, most procedural distress interventions continue to rely heavily on traditional CBT techniques (e.g., distraction, imagery, relaxation), with few studies testing newer, multimodal strategies. This gap is concerning given the increasing emphasis on a biopsychosocial treatment approach in pediatric oncology management (100). The integration of more contemporary, evidence-based behavioral interventions into pediatric cancer care could substantially enhance treatment outcomes and better address the complex evolving needs of patients.

The majority of behavioral interventions included in this review were implemented in isolation, rather than in combination with other behavioral domains. This is an important first step to establish evidence before moving to multi-component interventions. Indeed , very few studies integrated multi-component approaches. Exceptions included the small number of interventions that combined physical activity with CBT or dietary counseling, which demonstrated potential for broader quality-of-life improvements (55). However, these approaches remain the minority, highlighting an area for potential growth in future research and clinical application.

Promising next steps in this field include tailoring and personalizing interventions, examining the impact of behavioral interventions on health outcomes, and establishing specific guidelines for health behaviors during ALL treatment. First, a focus on tailoring and personalizing generalized health behavior interventions for youth with ALL may be useful to increase feasibility and acceptability to move the field toward efficacy testing. Models such as the Obesity-Related Behavioral Intervention Trials (ORBIT) model for behavioral treatment development (101) and the Framework for Reporting Adaptations and Modifications-Enhanced (FRAME) (102) could be particularly useful for adapting interventions to specific types of cancer (such as ALL), phases of treatment, or subpopulations with unique needs. Conducting RCTs to test these tailored interventions is crucial, as it helps overcome feasibility concerns and assess effectiveness. A second important area is examining the impact of behavioral interventions on health outcomes. Most studies to date have concentrated on behavioral outcomes, but it is well-established that these behavioral changes can lead to improved cancer outcomes and overall health. Recent literature highlights the significant role of health behaviors, including nutrition, activity, and sleep in influencing cancer outcomes, such as treatment tolerability and relapse rates (103, 104). However, very few studies have measured these effects directly (91). Finally, the development of established guidelines for health behaviors in pediatric ALL would provide clinically meaningful intervention targets. For physical activity, the International Physical Activity and Exercise Guidelines for youth with cancer–developed Delphi/roundtable consensus approach–provide a useful framework (105). However, widely established guidelines for nutrition, sleep, and other behaviors in the pediatric ALL context are lacking, aside from general recommendations based on age and developmental stage. Addressing these gaps and integrating comprehensive, individualized health behavior interventions could enhance measurement of intervention efficacy, as well as quality of life and long-term outcomes for pediatric cancer patients.

While the current review has multiple strengths, such as the use of multiple coders for data screening and extraction, extraction from multiple databases, and establishment of search terms within each database, findings should be interpreted in the context of several limitations. First, the results of this scoping review may be subject to publication bias as grey literature sources, such as unpublished and non-peer reviewed research (e.g., dissertations, book chapters), were excluded. These sources often contain valuable information, including studies with null or inconclusive results that are less likely to be published in traditional peer-reviewed journals. The exclusion of these types of sources may have resulted in the unintentional omission of behavioral interventions used in pediatric ALL that were not captured in the peer-reviewed literature. Second, while scoping reviews are by design useful for mapping the extent of literature, such as providing a general overview of the types of behavioral interventions implemented during active pediatric ALL treatment, they do not provide the same in-depth analysis or synthesis of evidence as systematic reviews and meta-analyses. As such, additional research is needed to examine the effectiveness or impact of these interventions on the outcomes included in this review. Additionally, the inclusion of studies with diverse methodologies and outcomes may have led to challenges in capturing the finer nuances or subtle findings in the literature. This review aimed to map outcomes of behavioral interventions for children receiving treatment for acute lymphoblastic leukemia. Explicit inclusion of these interventions were subsequently incorporated into the review’s search, and did not necessarily capture novel interventions or more generalized behavior interventions such as physical activity. While this does limit the applicability of the review’s data and analysis to anticipated interventions it still provides a useful mapping of evidence and information that may help inform the design and conduct of subsequent mapping and synthesis. Finally, the variability in study designs, participant characteristics, and intervention types may limit the generalizability of the findings, and the lack of a formal quality assessment of the included studies further reduces the certainty of the conclusions drawn.

The current state of the literature on behavioral health promotion interventions during pediatric ALL treatment is broad and lacks depth in multiple areas. While significant attention has been given to interventions targeting physical activity/exercise and procedural distress, the body of research is constrained by small sample sizes and a tendency for studies to stall at the feasibility stage, limiting the progression to efficacy testing. As a result, relatively few RCTs have been conducted in this field. These findings underscore the need for further research to strengthen the evidence base, particularly through larger, more rigorous trials that assess the effectiveness of behavioral interventions on a broader range of outcomes for children undergoing ALL treatment. Advancing behavioral health research, coupled with more rigorous efficacy testing, will be essential for enhancing the quality of care and improving both immediate and long-term psychological and physical outcomes for pediatric ALL patients.
